# MGST3 defines a mitochondrial-health-associated M2 macrophage state in atherosclerosis: integrative multi-omics, single-cell mediation, and functional validation

**DOI:** 10.3389/fimmu.2026.1864957

**Published:** 2026-09-17

**Authors:** Qiong-Chao Zou, Yong-Hong Yi, Jin He, Jing-Jing Rong, Hong-Wei Pan, Peng-Fei Zheng

**Affiliations:** 1 Cardiology Department, Hunan Provincial People’s Hospital, Changsha Hunan, China; 2 Clinical Research Center for Heart Failure in Hunan Province, Changsha, Hunan, China; 3 Institute of Cardiovascular Epidemiology, Hunan Provincial People’s Hospital, Changsha, Hunan, China; 4 Department of Cardiology, The First Affiliated Hospital of Hunan Normal University (Hunan Provincial People’s Hospital), Changsha, Hunan, China; 5 School of Medicine, Hunan Normal University, Changsha, China

**Keywords:** atherosclerosis, M2 macrophage, mendelian randomization, MGST3, mitochondrial health, single-cell mediation

## Abstract

**Abstract Background:**

Mitochondrial dysfunction is increasingly recognized as a driver of maladaptive macrophage polarization in atherosclerosis (AS), but the mitochondrial genes that shape disease-relevant macrophage states remain poorly defined.

**Methods:**

We integrated weighted gene co-expression network analysis (WGCNA), machine learning, two-sample Mendelian randomization (two-sample MR), bulk transcriptomics, single-cell analysis, disease-level validation, single-cell mediation, and *in vitro* perturbation to identify macrophage-relevant mitochondrial regulators in AS.

**Results:**

Eight candidate genes were consistently prioritized by WGCNA and machine learning; among them, microsomal glutathione S-transferase 3 (MGST3) showed the strongest protective genetic signal. Two-sample MR supported a protective association between genetically predicted MGST3 expression and coronary artery disease/coronary heart disease risk (training set OR = 0.989, 95% CI 0.978-0.999, P = 0.042; test set OR = 0.977, 95% CI 0.960-0.995, P = 0.013). Human carotid plaque validation confirmed reduced MGST3 at both mRNA and protein levels in AS lesions. Notably, despite this whole-plaque downregulation, single-cell and disease-level analyses revealed an expanded MGST3-high M2-like macrophage state in AS, suggesting a compensatory protective macrophage program within diseased plaques. MGST3-high M2-like cells displayed an OXPHOS-oriented, antioxidant, and less inflammatory transcriptional state, acted as an active signaling population, and showed higher composite MitoHealth scores. Single-cell mediation analysis further indicated that MitoHealth statistically links higher MGST3 expression to lower inflammatory activity, lower M1-like polarization, and lower atherogenic macrophage programming. In M2-polarized macrophages, MGST3 knockdown reduced M2-associated and mitochondrial/antioxidant transcripts while increasing pro-inflammatory transcripts, providing transcript-level functional support. PheWAS analysis detected no significant phenotype associations at the predefined threshold.

**Conclusions:**

These findings define MGST3 as a marker of a mitochondrial-health-associated M2-like macrophage state that appears expanded within AS plaques despite reduced whole-plaque MGST3 expression. The MGST3-MitoHealth axis may represent a compensatory macrophage program linked to mitochondrial preservation and lower pro-atherogenic activation, warranting further diagnostic and therapeutic investigation in AS.

## Introduction

Atherosclerosis (AS) is a chronic inflammatory disease of the arterial wall in which lipid deposition, immune-cell recruitment, and vascular remodeling progressively generate plaques that may rupture and trigger acute ischemic events (1–3). Although early lesions can remain clinically silent, advanced plaques may undergo fibrotic, calcified, and necrotic remodeling before precipitating myocardial infarction or cerebral ischemia, both major contributors to global mortality (4, 5). Even with effective management of dyslipidemia, hypertension, diabetes, and other classical risk factors, substantial residual cardiovascular risk persists (6, 7). This residual burden underscores the need to define local, cell-type-specific molecular programs that operate within plaques and may inform earlier detection or more selective intervention.

Macrophage dysfunction is central to AS pathogenesis (8). Although plaque macrophages exist along a continuum of highly plastic states, the M1/M2 framework remains useful for studying polarization-dependent gene function. In this framework, lipopolysaccharide or interferon-gamma promotes inflammatory M1-like activation, whereas IL-4-induced M2-like macrophages are associated with lipid handling, efferocytosis, and tissue repair (9, 10). Monocytes and macrophages dominate the immune landscape across AS stages (10). After internalizing atherogenic lipoproteins, including low-density lipoprotein and lipoprotein(a), macrophages can become foam cells and shift toward inflammatory cytokine production, thereby reinforcing oxidative lipoprotein modification, lipid accumulation, and local inflammation (11, 12). Although immune infiltration and mitochondrial dysfunction have both been implicated in AS progression (13), the mitochondrial genes that shape macrophage polarization within plaques remain insufficiently resolved.

Mitochondria govern macrophage polarization and effector function. Inflammatory M1-like macrophages rely predominantly on glycolysis and generate abundant reactive oxygen species, whereas reparative M2-like macrophages are more dependent on oxidative phosphorylation and fatty acid oxidation (14). Because M2-like polarization is metabolically coupled to oxidative metabolism, mitochondrial genes are likely to exert especially relevant effects within this compartment, making polarization-resolved analysis essential (14). Failure of mitochondrial quality control, whether through excessive mitochondrial ROS, defective mitophagy, or altered fission-fusion dynamics, can push macrophages toward inflammatory activation and tissue injury (15–18). However, which mitochondrial genes couple redox metabolism, lipid stress, and macrophage polarization within AS plaques remains unclear.

Most bioinformatics studies have prioritized mitochondrial genes from bulk-tissue correlations (19), an approach that cannot determine whether a candidate gene acts within a specific macrophage state or whether its association with disease is genetically supported. This limitation is particularly important in AS plaques, where whole-tissue expression profiles average signals across vascular, stromal, and immune populations and may therefore obscure adaptive or compensatory macrophage subpopulations that emerge within diseased lesions. To address this gap, we integrated WGCNA, machine learning, and two-sample Mendelian randomization to prioritize mitochondrial genes and test whether genetically predicted expression was associated with CAD/CHD risk. This unbiased framework prioritized microsomal glutathione S-transferase 3 (MGST3), a MitoCarta-annotated member of the membrane-associated proteins in eicosanoid and glutathione metabolism (MAPEG) family, as the most consistent candidate (20, 21). The biological position of MGST3 at the intersection of glutathione-dependent redox buffering and lipid-mediator metabolism (22) made it particularly plausible in the lipid-rich and oxidative plaque environment. We then used single-cell transcriptomics (23) and disease-level signature validation to map the cellular context of MGST3, followed by computational perturbation, single-cell mediation, human carotid plaque validation, and *in vitro* knockdown to evaluate its functional relevance in M2-like macrophages. This framework defined an MGST3-high M2-like macrophage state linked to mitochondrial integrity, lower inflammatory activation, and potential plaque-protective function in AS, while highlighting the need for future activity-based and *in vivo* validation.

## Materials and methods

### Study datasets, preprocessing, and genetic instruments

Datasets GSE28829, GSE41571, GSE43292, and GSE163154 (147 samples in total) were obtained from the Gene Expression Omnibus (GEO; http://www.ncbi.nlm.nih.gov/geo), integrated into a discovery expression matrix, and used for candidate screening, immune infiltration, and disease-level analyses. These cohorts represent related but non-identical AS phenotypes, including advanced versus early lesions, ruptured versus stable plaques, plaque versus macroscopically intact arterial tissue, and intraplaque hemorrhage versus non-hemorrhage. Accordingly, the integrated matrix was treated as a discovery framework rather than a single homogeneous clinical cohort. Three additional GEO datasets (GSE97320, 3 acute myocardial infarction and 3 control samples; GSE226790, 3 advanced atherosclerotic plaques and 3 healthy samples; GSE104140, 13 control and 19 AS samples) were combined with the discovery cohorts in a seven-cohort meta-analysis of the MGST3-high M2 signature. Single-cell RNA-seq data (GSE159677; 3 healthy and 3 AS samples) were used for macrophage polarization and mechanistic analyses, and GSE111782 (9 atherosclerotic and 9 control carotid samples) served as an independent cohort for signature and mitochondrial-module co-expression validation. Bulk datasets were processed according to data type before integration. For bulk microarray datasets, probe-level expression matrices were processed using limma (24); probes mapping to multiple genes were excluded, and multiple probes mapping to the same gene were averaged. For bulk RNA-seq datasets, gene-level expression matrices were processed using normalized expression values or count-based library-size normalization before downstream analysis. Batch effects across the four discovery bulk datasets were corrected using ComBat from the sva package before matrix integration. Single-cell RNA-seq data were processed separately using a Seurat-based workflow and were not included in the ComBat-corrected bulk matrix. Robustness across heterogeneous bulk datasets was assessed using cohort-stratified analyses, random-effects meta-analysis, leave-one-out sensitivity analysis, and independent validation in GSE111782 where appropriate. All statistical analyses were performed in R (version 4.5.2). Two-sided P < 0.05 was considered statistically significant, and Benjamini-Hochberg FDR correction was used where appropriate.

Gene expression instruments were obtained from the eQTLGen consortium (25) and the Genotype-Tissue Expression (GTEx) V8 project (26), which provided sample sizes of 31,684 and 755, respectively, for identifying SNPs associated with target-gene expression. GWAS summary statistics for cardiovascular outcomes were then used to assess the genetic association between mitochondrial-gene expression and disease risk. CAD summary data (34,541 cases and 261,984 controls; OpenGWAS ID: ebi-a-GCST005194) were used as the test set, and CHD summary data (60,801 cases and 123,504 controls; OpenGWAS ID: ieu-a-7) were used as the validation set; both were accessed through the MRC IEU OpenGWAS data infrastructure (27) (https://opengwas.io/datasets/). Because all GEO and GWAS datasets were publicly available and generated under the ethical approvals and consent procedures of the original studies, no additional ethical approval or informed consent was required for these secondary analyses.

### Identification of key mitochondrial genes by WGCNA and machine learning

WGCNA was performed on the 25% most variable genes in the integrated discovery matrix (28), following our previously described protocol (29). A soft-thresholding power of 12 was selected to approximate scale-free topology, and AS-related co-expression modules were identified. Genes from the trait-associated module were intersected with 1,136 MitoCarta3.0-annotated mitochondrial genes (20), yielding 51 candidates. These genes were prioritized using an established feature-selection workflow comprising LASSO logistic regression (glmnet) (30) and SVM-RFE (31), which selected 27 and 10 genes, respectively. Because machine learning was used for candidate prioritization rather than for deployment of a diagnostic classifier, downstream evidence from Mendelian randomization, single-cell analysis, disease-level validation, and functional assays was used to prioritize MGST3. The eight genes identified by both machine-learning methods were retained for subsequent two-sample Mendelian randomization analysis.

### Two-sample Mendelian randomization analysis

To test whether genetically predicted expression of the candidate mitochondrial genes was associated with CAD/CHD risk, we performed two-sample Mendelian randomization (32) using blood cis-eQTL instruments (25) and GWAS summary statistics for CAD (test set; ebi-a-GCST005194) and CHD (validation set; ieu-a-7) (27). Candidate instruments were cis-acting SNPs within ±1 Mb of each gene’s transcription start site; SNPs were clumped for linkage disequilibrium (r² < 0.001 within a 10 Mb window using the 1000 Genomes European reference panel) and retained when the F-statistic exceeded 10. Effects were estimated by inverse variance weighted regression for multi-SNP instruments and by the Wald ratio for single-SNP instruments, and are reported as odds ratios with 95% confidence intervals. Heterogeneity was assessed using Cochran’s Q, and horizontal pleiotropy was tested using the MR-Egger intercept (**Supplementary Table S7**). Six of the eight candidate genes (PPIF, PMAIP1, MRPL33, GATM, BCL2A1, and MGST3) had eligible cis-eQTL instruments; IMMP2L and CPS1 were excluded because no eligible blood cis-eQTLs were available. Because the instruments were blood-derived rather than plaque macrophage- or vascular tissue-specific, the MR results were interpreted as supportive genetic evidence rather than definitive proof of plaque-cell-specific causality.

### Immune cell infiltration analysis using CIBERSORTx

Immune infiltration was estimated with CIBERSORTx (https://cibersortx.stanford.edu/index.php) to characterize immune-microenvironment differences between AS and control/non-AS tissues. The analysis was performed on the integrated discovery dataset comprising GSE28829, GSE41571, GSE43292, and GSE163154. A total of 1,000 permutations was used to obtain stable estimates of relative immune-cell abundance, and the resulting profiles were visualized to summarize the AS immune landscape.

### Single-cell data processing and annotation

Single-cell RNA-seq data from GSE159677 (3 healthy and 3 AS carotid samples) were processed with Seurat (v5.4.0) (33). Cells with fewer than 100 detected genes and genes detected in fewer than three cells were removed. After normalization and highly variable gene selection, PCA and Harmony integration (34) were performed, and the first 20 principal components were used for clustering. Clusters were annotated into nine major cell types using CellMarker and published marker sets. Macrophage states were annotated as M1-like or M2-like rather than as fixed binary lineages, reflecting the continuum of plaque macrophage phenotypes. M1-like annotation was supported by pro-inflammatory markers and modules, including IL1B, TNF, CXCL10, CD80, CD86, NFKB1, and related inflammatory scores, whereas M2-like annotation was supported by reparative, lipid-handling, and scavenger-receptor markers, including MRC1, CD163, MSR1, MERTK, C1QA, C1QB, APOE, and related M2 module scores. MGST3 expression was visualized on UMAP projections and compared between M1-like and M2-like macrophages in the integrated dataset and within control and AS samples, thereby establishing the basis for MGST3-focused functional analyses in AS M2-like macrophages.

### Single-cell functional, communication, and mediation analyses

Unless otherwise stated, single-cell analyses were restricted to AS M2-like cells (n = 6,419). Cells with normalized MGST3 expression > 0 were considered MGST3-expressing; when >85% of AS M2-like cells expressed MGST3, the top and bottom tertiles defined MGST3-high and MGST3-low cells, and otherwise expressing versus non-expressing cells were compared. Functional module scores (OXPHOS, TCA cycle, mitochondrial translation, antioxidant/ROS, mitophagy, and inflammation) were calculated with AddModuleScore and compared between MGST3-high and MGST3-low cells by paired t-tests across the three AS samples; cell-level Spearman correlations with MGST3 were also computed. Expression of curated mitophagy-related genes (PINK1, BNIP3, BNIP3L, FUNDC1, OPTN, SQSTM1, MAP1LC3B, ATG5, ATG7, MFN1, MFN2, OPA1, PRKN, CALCOCO2) was also compared between MGST3-high and MGST3-low M2-like cells. A composite MitoHealth score was defined within AS M2-like cells as the mean of z-scored OXPHOS, TCA, mitochondrial translation, and antioxidant scores minus the z-scored inflammatory score, and was compared between groups and correlated with MGST3. The MGST3-high M2 signature was constructed from genes differentially expressed between MGST3-high and MGST3-low M2 cells (Wilcoxon rank-sum test; genes with adjusted P < 0.05 were ranked, and the top 100 genes, 50 up and 50 down, excluding MGST3, were retained) and scored in bulk samples as the difference between the mean z-scores of up- and down-regulated genes.

Pathway and transcription factor activities were compared using PROGENy (35) and DoRothEA (36) with weighted mean scoring; paired t-tests were applied across the three AS samples. Virtual knockdown of MGST3 was performed with scTenifoldKnk (37) (300 cells, 2,000 genes, three networks); genes were ranked by |Z| for visualization, and those with |Z| > 2 and adjusted P < 0.05 were subjected to GO biological process enrichment. These virtual knockdown results were treated as computational predictions of network perturbation rather than as direct loss-of-function evidence. Cell-cell communication was inferred with CellChat (v2) (38) using the triMean method and interactions with P < 0.05; signaling strengths, top ligand-receptor interactions, and pathway-level differences between MGST3-high and MGST3-low groups were summarized, and a group-label permutation analysis was performed as a sensitivity check (**Supplementary Table S10**). As a complementary bulk analysis, MGST3 co-expression was assessed in the 147-sample integrated discovery matrix using Spearman correlation with Benjamini-Hochberg FDR correction; co-expression with top OXPHOS genes was visualized, the top 50 co-expressed genes were tested for hypergeometric enrichment within MitoCarta-derived mitochondrial modules, and bootstrap resampling (100 iterations) was used to assess the stability of the top-50 co-expression network.

Disease-level evidence was evaluated through complementary bulk analyses. M2-like scores were estimated by ssGSEA (38) (alpha = 0.25) in the four discovery datasets. BisqueRNA deconvolution was then performed using single-cell-derived macrophage state markers, followed by random-effects meta-analysis of resident-like and total M2 proportions. The MGST3-high M2 signature was tested against M2-like proportions across seven bulk cohorts using metafor, with leave-one-out analysis used to assess robustness. GSE111782 was used for independent validation of MGST3 co-expression with the MGST3-high M2 signature and mitochondrial modules. Finally, single-cell mediation analysis tested whether MitoHealth mediated the association between MGST3 and pro-atherogenic macrophage phenotype scores (inflammatory, M1-like, and atherogenic composite). Path coefficients were estimated with linear mixed models (lme4) (39), using sample as a random effect and z-scored nFeature_RNA as a covariate. Total, direct, and indirect effects were decomposed, and indirect effects were estimated from 1,000 sample-cluster bootstrap resamples with percentile confidence intervals (40). Robustness was assessed by re-running the single-cell pipeline with an independent configuration, including SCTransform normalization, alternative QC thresholds, scDblFinder doublet removal, Harmony integration by sample, and marker-module-score-based cell-state annotation.

### Phenome-wide association study analysis

To determine whether genetically predicted MGST3 expression was associated with a broad range of human phenotypes, we performed PheWAS using the AstraZeneca PheWAS Portal (https://azphewas.com/) (41). The portal integrates gene-phenotype associations derived from electronic health records, questionnaires, and continuous traits computed from UK Biobank exomes, with genomic coordinates based on GRCh38. Continuous phenotypes were rank-based inverse-normal transformed before analysis. A significance threshold of P < 1E-8, consistent with the portal default, was applied to reduce false-positive findings. These analyses were interpreted as phenotype-association screening and not as evidence of pharmacological safety.

### Clinical sample collection

Carotid artery tissues were collected from ten patients undergoing endarterectomy for stenosis at Hunan Provincial People’s Hospital. For each patient, paired specimens were obtained from the atherosclerotic plaque and from macroscopically intact non-plaque distal intima within the same surgical sample. All tissues were snap-frozen in liquid nitrogen immediately after collection and stored at -80 °C until analysis. The study protocol was approved by the Medical Ethics Committee of Hunan Provincial People’s Hospital (Approval No. 2025-348), and written informed consent was obtained from all participants before tissue collection.

### *In vitro* macrophage culture, M2 polarization, and MGST3 knockdown

Human THP-1 monocytes (ATCC) were maintained in RPMI-1640 medium supplemented with 10% fetal bovine serum (FBS) and 1% penicillin-streptomycin at 37 °C in a humidified 5% CO2 incubator. Cells were differentiated into macrophages by treatment with 100 ng/mL phorbol 12-myristate 13-acetate (PMA) for 48 h. After medium replacement, macrophages were transfected with 50 nM non-targeting control siRNA (siNC) or MGST3-targeting siRNA (siMGST3) using Lipofectamine RNAiMAX (Thermo Fisher Scientific) according to the manufacturer’s instructions. After 24 h, M2 polarization was induced with recombinant human IL-4 and IL-13 (20 ng/mL each) for 48 h, and cells were harvested 72 h after transfection. Total RNA was extracted, reverse transcribed, and analyzed by RT-qPCR as described in the RT-qPCR section. The expression of MGST3, MRC1, CD163, IL10, IL1B, TNF, NDUFC1, COX7A2, GPX4, and SOD2 was normalized to ACTB (β-actin), with M0 cells as the reference (M0 = 1). Three biological replicates were analyzed per group (M0, M2 + siNC, and M2 + siMGST3). Knockdown efficiency greater than 50% was required, and polarization success was confirmed by elevated MRC1 and CD163 in M2 + siNC compared with M0 cells. Data are presented as mean ± SD; siNC versus M0 and siMGST3 versus siNC were compared by unpaired Welch t-test, with P < 0.05 considered significant.

### Quantitative real-time polymerase chain reaction

Total RNA was extracted from macroscopically intact arterial tissues, carotid plaques, and cultured cells using TRIzol reagent (Invitrogen, USA). First-strand cDNA was synthesized with the PrimeScript RT Reagent Kit (Takara Bio, Japan). RT-qPCR was performed on an ABI Prism 7500 Sequence Detection System (Applied Biosystems, USA) using 2x RealStar Fast SYBR qPCR Mix (Kangrun, Beijing, China). Target-gene expression was normalized to ACTB (beta-actin), and relative expression was calculated using the 2^-DeltaDeltaCt method. All samples were analyzed in technical triplicate, and primer sequences are provided in **Supplementary Table S14**.

### IHC staining

For IHC staining, paraffin sections were dewaxed, rehydrated, and subjected to heat-induced antigen retrieval in 0.1 M citrate buffer at 94 °C for 20 min, followed by cooling to room temperature. Endogenous peroxidase activity was blocked with 3% hydrogen peroxide for 25 min in the dark. After blocking with 3% bovine serum albumin for 30 min, sections were incubated overnight at 4 °C with primary antibody against MGST3 (Abcam, USA). Sections were then washed, incubated with goat anti-rabbit IgG-HRP (Solarbio, Beijing, China) for 90 min, developed with DAB, and counterstained with hematoxylin. Images were captured using an Olympus BX63 microscope and quantified with ImageJ (NIH, USA).

### Western blotting

Tissues were lysed in RIPA buffer supplemented with protease inhibitors, and protein concentrations were measured using a BCA kit (Thermo Fisher Scientific). Protein samples were denatured, separated by SDS-PAGE, and transferred to polyvinylidene fluoride membranes (EMD Millipore). Membranes were blocked with 5% skim milk for 2 h at room temperature, incubated overnight at 4 °C with primary antibodies, and then incubated with secondary antibodies for 2 h. Signals were detected using a Tanon chemiluminescence imaging system and quantified with ImageJ (NIH, USA).

## Results

### WGCNA identified an AS-associated co-expression module

After outlier removal, format harmonization, missing-value imputation, and batch correction, the GSE28829, GSE41571, GSE43292, and GSE163154 datasets were merged into an integrated discovery expression profile containing 12,638 unique gene symbols from 147 samples. The top 25% most variable genes were retained for WGCNA and are listed in **Supplementary Table S1**. A soft-thresholding power of 12 was selected, yielding an approximately scale-free network with a correlation coefficient greater than 0.8 (**Figure 1A**). Gene-expression correlations were converted into an adjacency matrix and then into a topological overlap matrix (TOM), which was used to relate co-expression structure to clinical traits across all samples (**Figure 1B**). Hierarchical average-linkage clustering based on TOM-derived dissimilarity generated the gene dendrogram (**Figure 1C**), and dynamic tree cutting separated genes with similar connectivity patterns into three modules (**Figure 1D**). Among these modules, the pink module showed the strongest trait associations, including ruptured stage (r = 0.83, P = 2E-38), advanced stage (r = 0.81, P = 2E-35), intraplaque hemorrhage (r = 0.79, P = 2E-32), and AS status (r = 0.54, P = 2E-12; **Figure 1E**). Within this module, module membership correlated strongly with gene significance (r = 0.66, P = 6.2E-161; **Figure 1F**), supporting the pink module as the primary AS-associated co-expression module for downstream mitochondrial-gene prioritization. Gene symbols, AS-related gene significance values, and corresponding P values for the pink module are provided in **Supplementary Table S2**.

**Figure 1:**
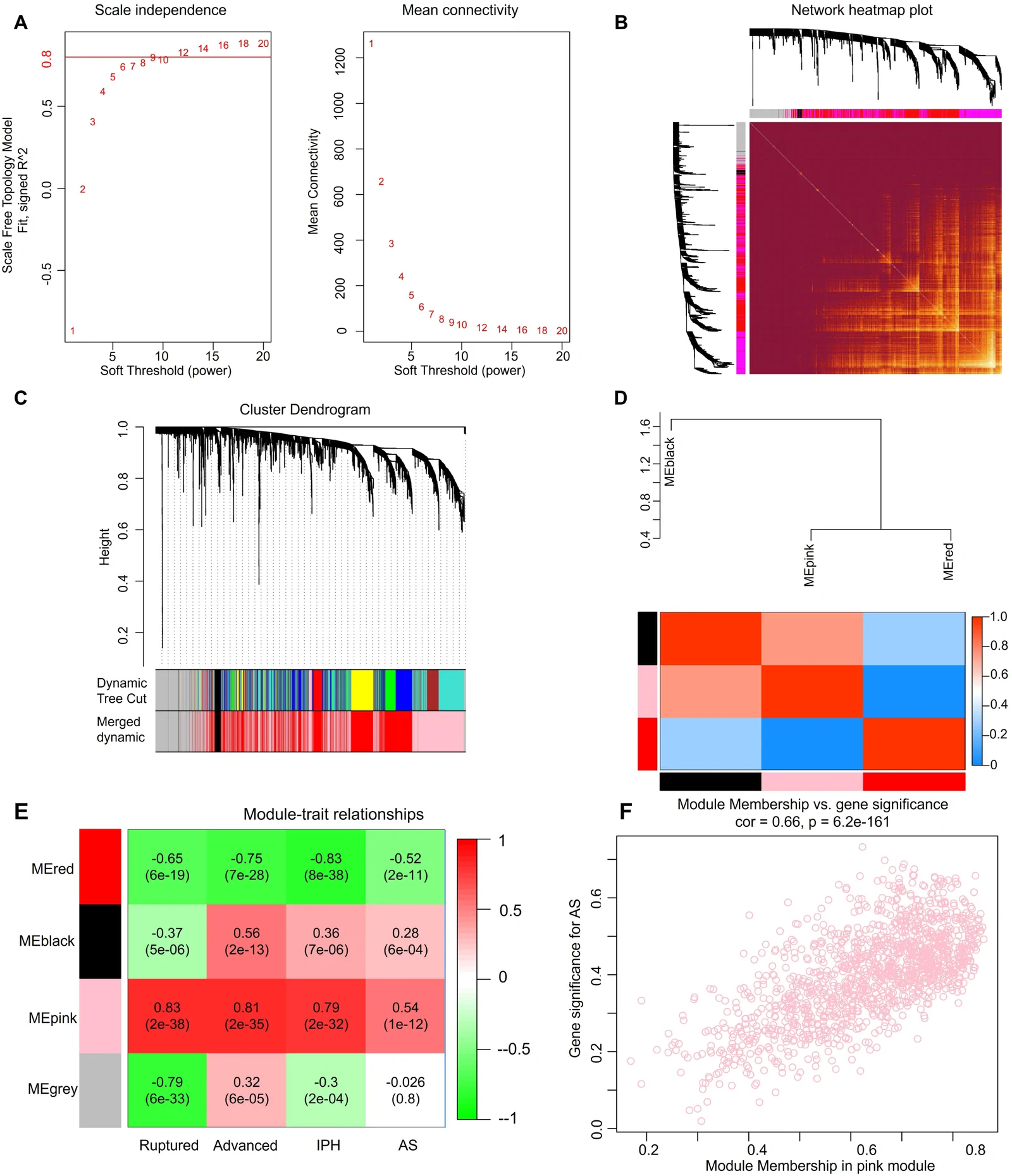
Weighted gene co-expression network analysis. **(A)** Analysis of network topology across candidate soft-thresholding powers. **(B)** Representative topological overlap matrix (TOM) heatmap of the gene co-expression network. **(C)** Gene clustering dendrogram based on TOM-derived dissimilarity. **(D)** Module assignment by dynamic tree cutting. **(E)** Correlations between gene modules and clinical traits. **(F)** Association between gene significance and module membership in the pink module.

### Identification of key mitochondrial genes by machine learning and two-sample MR validation

Intersecting the 1,280 AS-associated pink-module genes with 1,136 mitochondrial genes (**Supplementary Table S3**) identified 51 mitochondrial genes within the pink module (**Figure 2A**; **Supplementary Table S4**). LASSO regression prioritized 27 of these genes (**Figure 2B**), whereas SVM-RFE prioritized 10 (**Figure 2C**). Eight genes were selected by both algorithms and retained for downstream analysis: inner mitochondrial membrane peptidase subunit 2 (IMMP2L), BCL2-related protein A1 (BCL2A1), glycine amidinotransferase (GATM), peptidylprolyl isomerase F (PPIF), phorbol-12-myristate-13-acetate-induced protein 1 (PMAIP1), mitochondrial ribosomal protein L33 (MRPL33), carbamoyl-phosphate synthase 1 (CPS1), and microsomal glutathione S-transferase 3 (MGST3) (**Figure 2D**; **Supplementary Table S5**).

**Figure 2:**
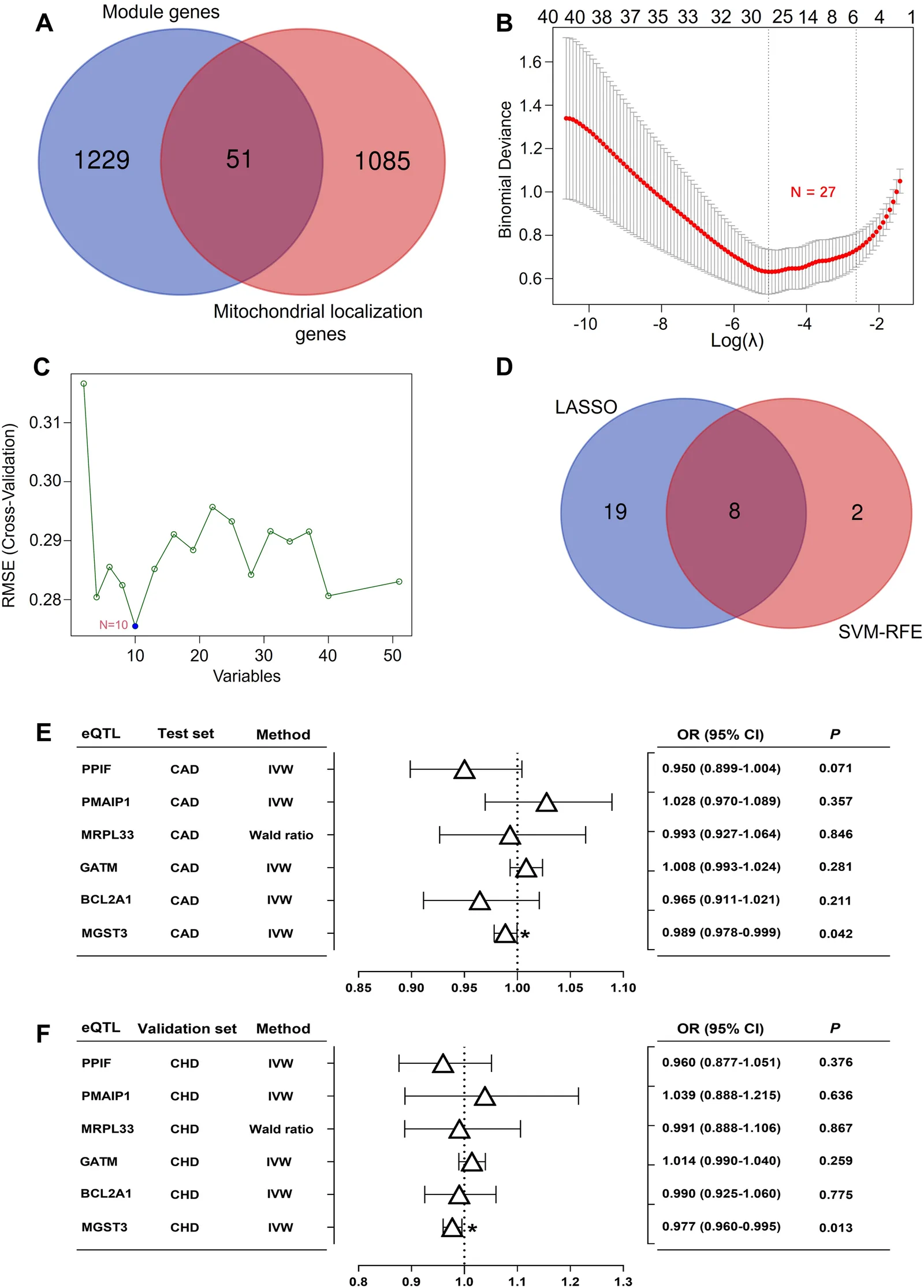
Identification of key mitochondrial genes by machine learning and two-sample MR validation. **(A)** Venn diagram showing the intersection of 1280 pink-module genes and 1136 mitochondrial genes. **(B)** Key genes identified by LASSO regression. **(C)** Key genes identified by the SVM-RFE algorithm. **(D)** Venn diagram of genes consistently identified by both machine learning methods. **(E, F)** Two-sample MR forest plot of six key mitochondrial genes with CHD risk in the test set **(E)** and validation set **(F)**; effect sizes are expressed as ORs with 95% CIs (IVW or Wald ratio).

Two-sample MR was used to test genetic associations between six instrument-eligible mitochondrial genes (PPIF, PMAIP1, MRPL33, GATM, BCL2A1, and MGST3) and CAD/CHD risk. The CAD test set and CHD validation set were analyzed separately to assess robustness (**Figures 2E**, **F**). MGST3 was consistently associated with reduced atherosclerotic cardiovascular disease risk in both datasets (CAD test set: OR = 0.989, 95% CI 0.978-0.999, P = 0.042; CHD validation set: OR = 0.977, 95% CI 0.960-0.995, P = 0.013), supporting a potential protective genetic association. No significant associations were detected for PPIF, PMAIP1, MRPL33, GATM, or BCL2A1. Detailed MR results are provided in **Supplementary Table S7**.

### Immune microenvironment characteristics between control and AS samples

Immune-infiltration analysis revealed clear differences between control/non-AS and AS samples (**Supplementary Figure S1A**; **Supplementary Table S6**). Compared with control specimens, AS samples showed higher inferred M0 macrophage abundance and lower infiltration of CD8 T cells and resting dendritic cells. Correlation analysis between the eight selected mitochondrial genes and M0 macrophage abundance further revealed divergent patterns (**Supplementary Figure S1B**). PPIF, PMAIP1, GATM, and BCL2A1 were positively correlated with M0 macrophage infiltration, whereas MRPL33, MGST3, IMMP2L, and CPS1 were negatively correlated with this immune-cell subset.

### Single-cell analysis localizes MGST3 to M2-like macrophages

After quality control, normalization, and dimensionality reduction of the six GSE159677 single-cell samples, unsupervised clustering identified 20 cell subpopulations (**Figure 3A**). Cluster marker genes were compared with CellMarker and published marker sets to assign cell identities (**Figure 3B**). Manual annotation resolved nine major cell populations: M1-like macrophages, M2-like macrophages, T cells, B cells, plasma cells, mast cells, natural killer cells, endothelial cells, and vascular smooth muscle cells (**Figure 3C**).

**Figure 3:**
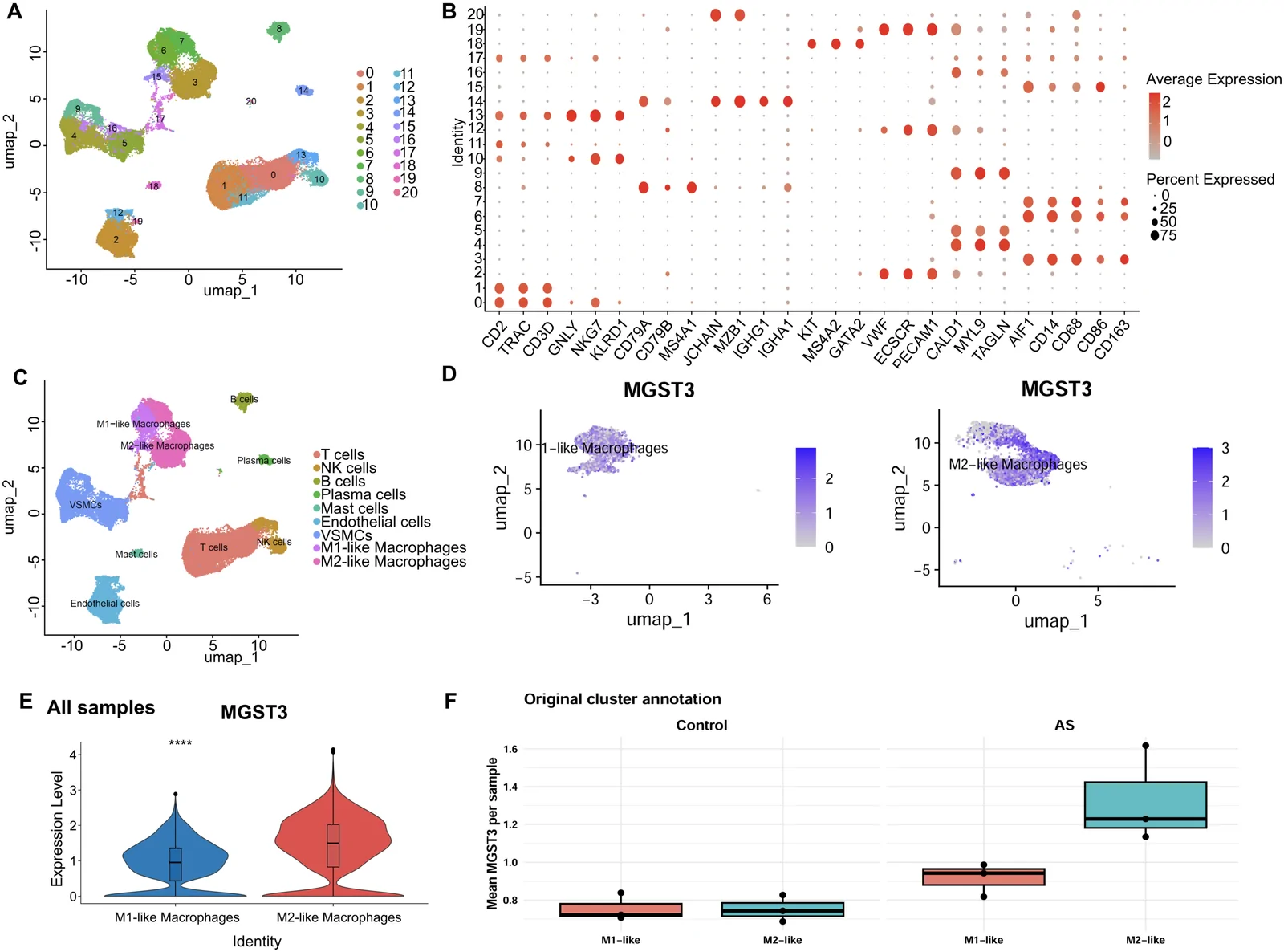
Single-cell analysis localizes MGST3 to M2-like macrophages. **(A)** Unsupervised clustering of 20 cell subpopulations in GSE159677. **(B)** Cell-type annotation based on known marker genes from CellMarker and published marker sets. **(C)** Final annotated cell populations, including M1-like macrophages, M2-like macrophages, T cells, B cells, plasma cells, mast cells, NK cells, endothelial cells, and VSMCs. **(D)** UMAP feature plot showing MGST3 expression in M1-like and M2-like macrophages. **(E)** MGST3 expression in M1-like versus M2-like macrophages in the integrated single-cell dataset. **(F)** MGST3 expression in M1-like versus M2-like macrophages stratified by control and AS samples ****P < 0.0001..

MGST3 expression was then mapped across macrophage subsets. UMAP visualization showed MGST3 expression in both M1-like and M2-like macrophages (**Figure 3D**), and quantitative comparisons demonstrated higher MGST3 expression in M2-like macrophages than in M1-like macrophages in the integrated dataset (**Figure 3E**). This M2-like enrichment was maintained in AS samples and showed a similar, although non-significant, trend in control samples (**Figure 3F**), indicating that MGST3 is preferentially associated with M2-like macrophage polarization under AS conditions.

### Functional state of MGST3-high M2 macrophages.

Having established that MGST3 is preferentially expressed in M2-like macrophages, we next asked whether MGST3-high M2-like cells represent a distinct functional state within AS plaques. MGST3-high M2-like cells showed higher OXPHOS (+0.113, paired P = 0.011), TCA cycle (+0.059, P = 0.0006), mitochondrial translation (+0.054, P = 0.009), and ROS/antioxidant defense (+0.201, P = 0.028) module scores, together with lower inflammatory scores (-0.073, P = 0.022; **Figure 4A**). These changes define a metabolically active, antioxidant, and less inflammatory M2-like state. Cell-level Spearman analysis confirmed positive associations between MGST3 and OXPHOS, TCA cycle, and ROS/antioxidant defense scores, and a negative association with the inflammatory score (**Figure 4B**), supporting a continuous relationship between MGST3 abundance and mitochondrial homeostasis. Mitophagy-related genes, including PINK1, BNIP3, BNIP3L, FUNDC1, OPTN, SQSTM1, MAP1LC3B, ATG5, ATG7, MFN1, MFN2, OPA1, PRKN, and CALCOCO2, were also elevated in MGST3-high M2-like cells (**Figure 4C**), suggesting enhanced mitochondrial quality-control activity. We then asked whether this phenotype was reflected at the bulk plaque-transcriptome level using the ComBat-corrected four-cohort discovery matrix. In this integrated bulk dataset, MGST3 was positively co-expressed with multiple OXPHOS genes, including NDUFC1, COX7A2, NDUFAB1, and COX7C (**Figure 4D**), and MGST3-associated genes were enriched for OXPHOS (P = 6.8E-4) and TCA cycle modules (**Figure 4E**). Bootstrap resampling showed that the core MGST3-associated co-expression genes were repeatedly retained among top-ranked genes across resampled datasets, indicating that the MGST3-mitochondrial co-expression pattern was stable and unlikely to be driven by a small subset of samples (**Figure 4F**). Together, these results support MGST3 as a marker of mitochondrial homeostasis and a less inflammatory metabolic state in M2-like macrophages, providing the functional basis for subsequent mechanistic and disease-level analyses. Detailed results are provided in **Supplementary Table S8**.

**Figure 4:**
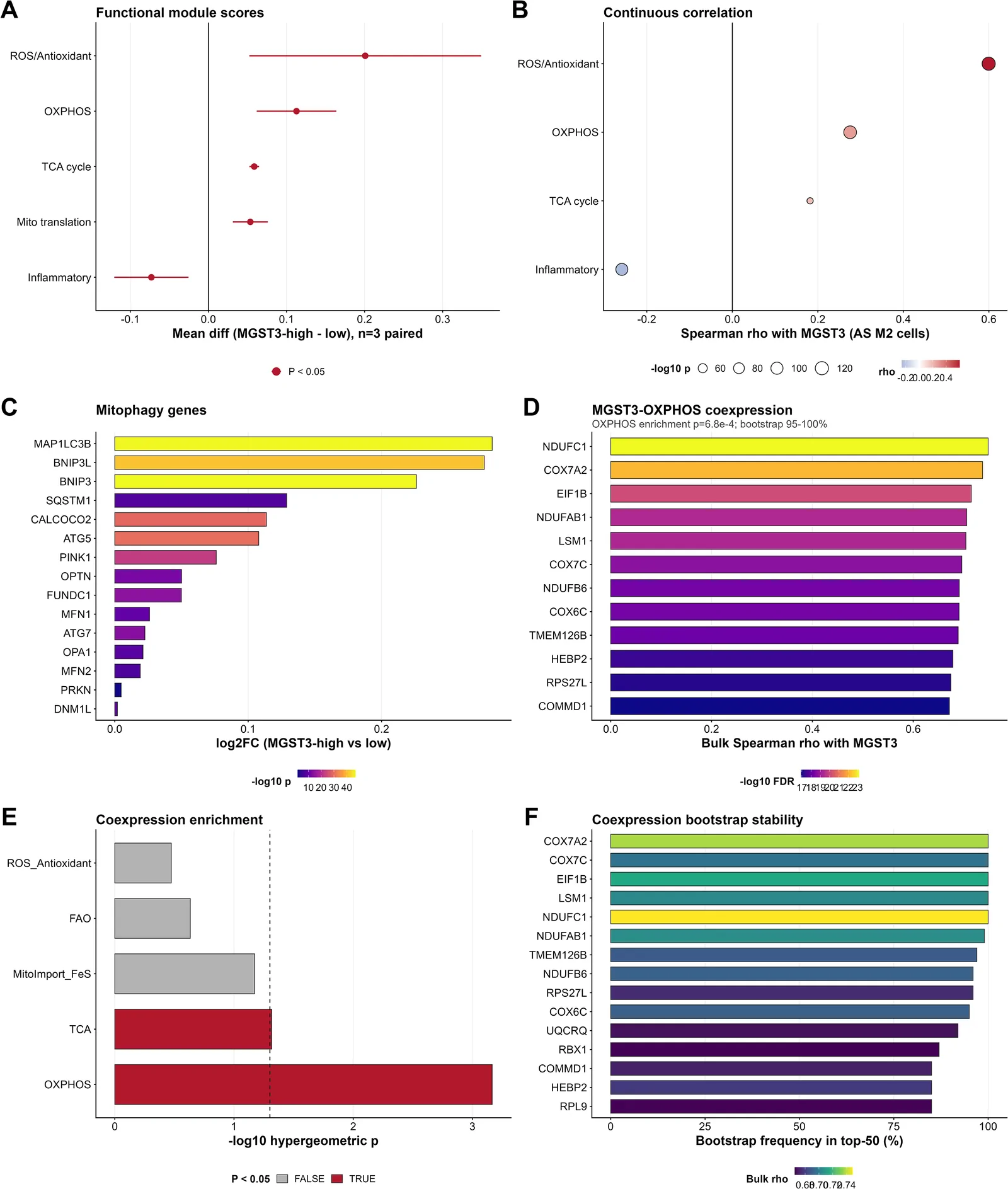
Functional state of MGST3-high M2-like macrophages. **(A)** Paired comparison of mitochondrial and inflammatory module scores between MGST3-high and MGST3-low M2-like cells across three AS samples. **(B)** Cell-level Spearman correlations between MGST3 and module scores. **(C)** Mitophagy-related genes in MGST3-high versus MGST3-low M2-like cells. **(D)** Bulk co-expression of MGST3 with top OXPHOS genes. **(E)** Hypergeometric enrichment of co-expressed genes in mitochondrial modules. **(F)** Bootstrap stability of the top-50 co-expression network.

### Molecular mechanism of MGST3 in M2 macrophages

To investigate molecular programs underlying the MGST3-associated mitochondrial homeostatic M2-like state, we compared inferred pathway and transcription-factor activities between MGST3-high and MGST3-low M2-like cells. PROGENy analysis showed lower Hypoxia, TGF-beta, and VEGF activities and higher p53 activity in MGST3-high M2-like cells (**Figure 5A**), consistent with reduced hypoxic stress, attenuated tissue-remodeling/pro-inflammatory signaling, and a more homeostasis-oriented regulatory state. Inflammatory transcription factors, including REL, STAT1, TBX21, NFATC1, ETS1, and RUNX1, also showed lower activity in MGST3-high M2-like cells (**Figure 5B**), indicating suppression of NF-kB/interferon- and inflammation-related transcriptional programs. To determine whether MGST3 might help maintain this state rather than merely mark it, we performed virtual MGST3 knockdown with scTenifoldKnk. Predicted MGST3 perturbation affected genes involved in lipid handling and inflammatory mediator metabolism, including FABP5, MIF, CSTB, TPI1, and S100A10 (**Figure 5C**). GO enrichment further highlighted unsaturated fatty acid and prostanoid metabolic processes (**Figure 5D**), linking MGST3 disruption to lipid-derived inflammatory mediator pathways. These findings suggest that MGST3 is associated not only with mitochondrial homeostasis but also with the transcriptional architecture and lipid-mediator metabolism that support a less inflammatory M2-like program. Detailed results are provided in **Supplementary Table S9**.

**Figure 5:**
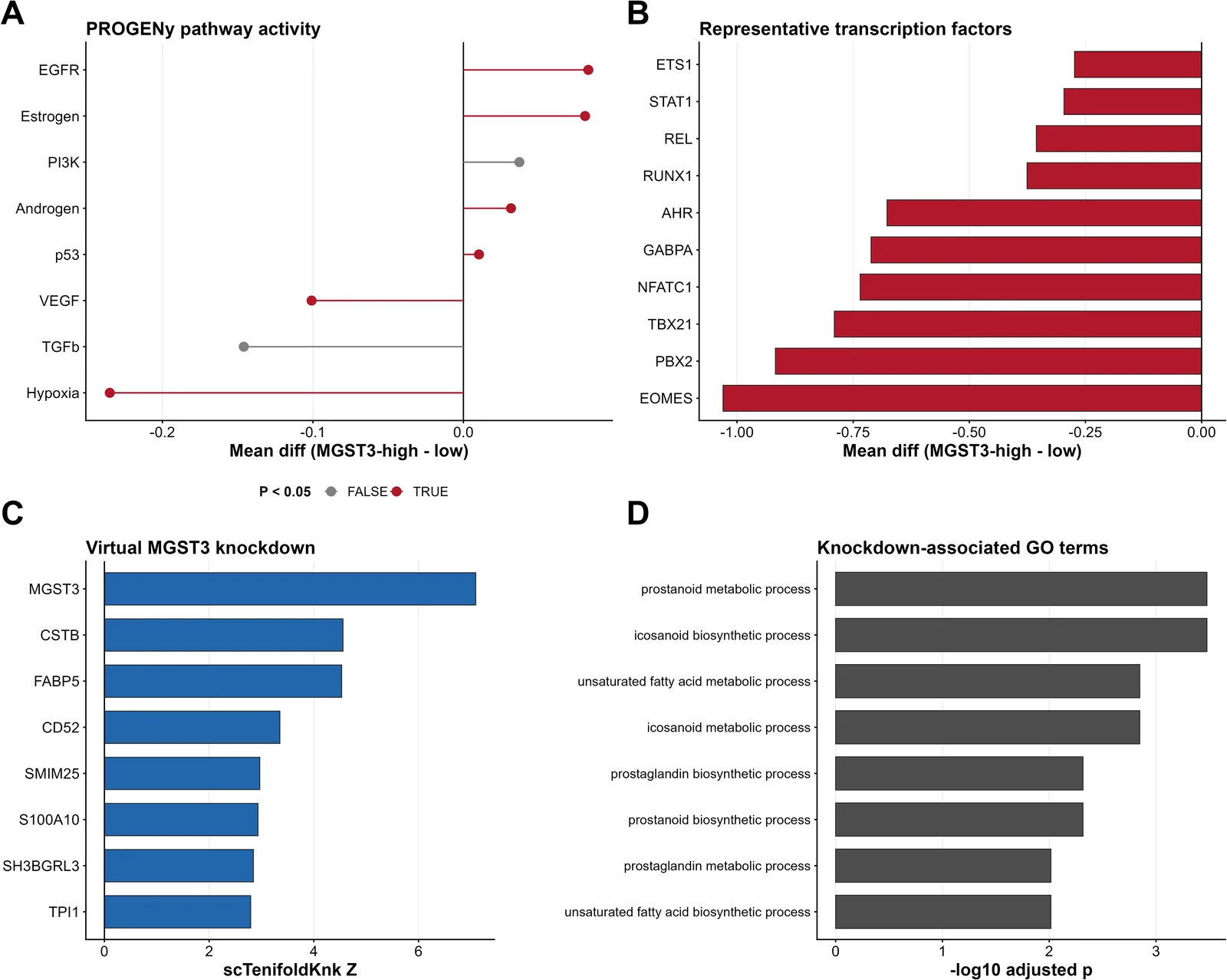
Molecular mechanism of MGST3 in M2-like macrophages. **(A)** PROGENy pathway activity differences between MGST3-high and MGST3-low M2-like cells. **(B)** Representative transcription factor activity differences. **(C)** Top genes perturbed by scTenifoldKnk virtual knockdown of MGST3. **(D)** GO biological process enrichment of perturbed genes.

### Cell-cell communication of MGST3-high M2 macrophages

To determine whether the MGST3-high mitochondrial homeostatic M2-like state was coupled to altered microenvironmental communication, we performed cell-cell interaction analysis within plaques. Compared with MGST3-low M2-like cells, MGST3-high M2-like cells showed higher outgoing signaling strength (4.405 vs 1.771), indicating stronger inferred signal-sending capacity, and higher incoming signaling strength (3.757 vs 1.801), indicating greater responsiveness to signals from surrounding plaque-resident cells (**Figure 6A**). These findings suggest that MGST3-high M2-like macrophages may act as a bidirectionally active communication hub within the plaque immune niche. Incoming interactions were dominated by CD99-CD99, MIF-CD74/CD44, MHC-II-CD4, and SPP1-CD44 (**Figure 6B**), suggesting exposure to adhesion-, MIF-, antigen-presentation-, and SPP1-related cues. Outgoing interactions were enriched for SPP1-CD44, MIF-CD74/CXCR4, CD99-CD99, and LGALS9-CD45 (**Figure 6C**), implicating these cells in tissue-remodeling, immune-regulatory, adhesion, and galectin-mediated signaling. At the pathway level, SPP1, MIF, CD99, MHC-II, and GALECTIN were the dominant communication programs enriched in MGST3-high M2-like cells (**Figure 6D**), indicating that the phenotype was organized beyond isolated ligand-receptor pairs. Together, these findings suggest that MGST3-high M2-like macrophages may coordinate immune-regulatory, antigen-presentation, adhesion, and tissue-remodeling signals within the plaque microenvironment. Detailed results and permutation-based robustness analyses are provided in **Supplementary Table S10**.

**Figure 6:**
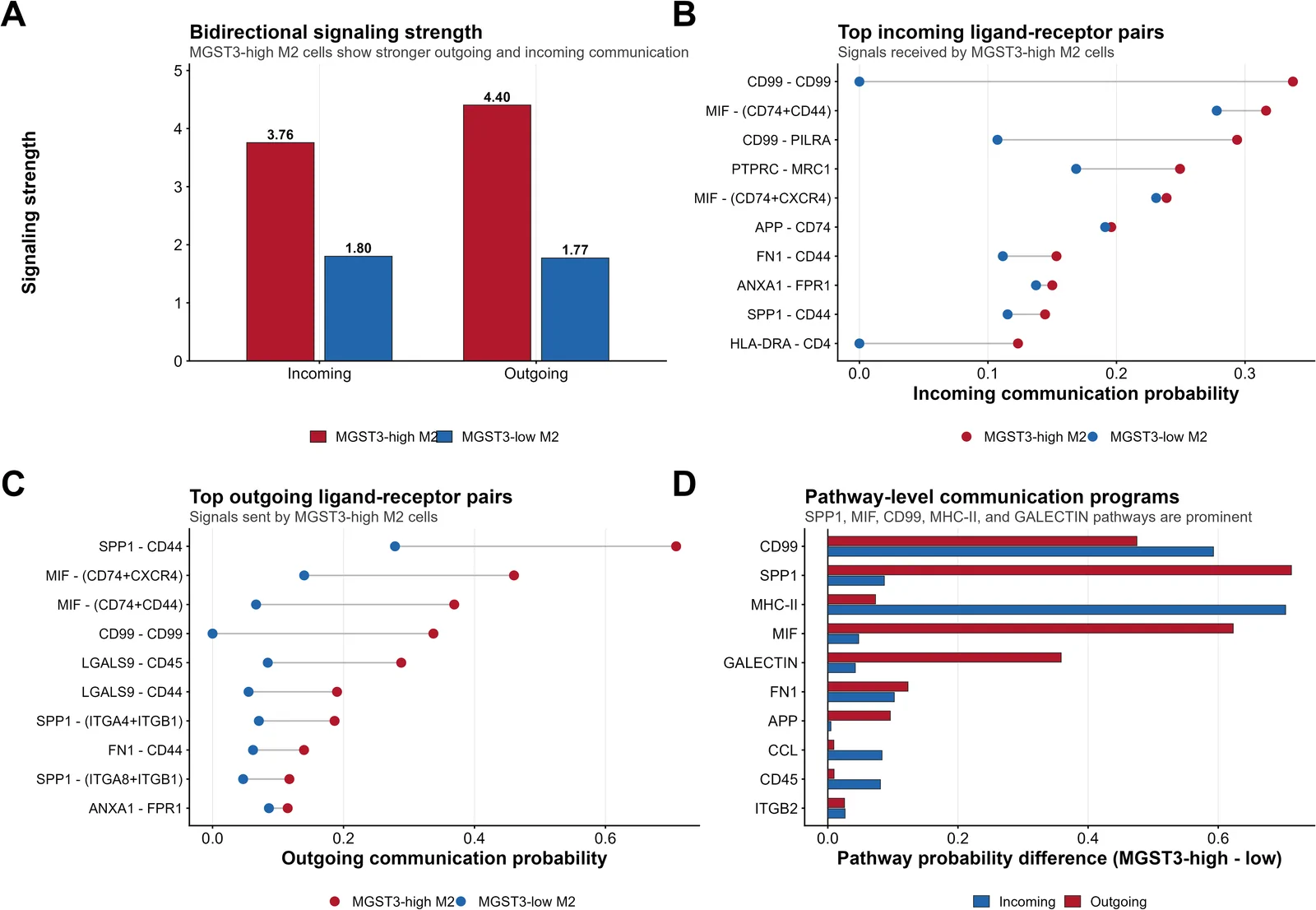
Cell-cell communication of MGST3-high M2-like macrophages. **(A)** Incoming and outgoing signaling role strengths of cell populations, highlighting the signaling activity of MGST3-high M2-like cells. **(B)** Top incoming ligand-receptor interactions to MGST3-high M2-like cells. **(C)** Top outgoing ligand-receptor interactions from MGST3-high M2-like cells. **(D)** Top communication pathways differentially enriched in MGST3-high versus MGST3-low M2-like cells.

### Disease-level bulk validation of the MGST3-high M2 state

To determine whether the single-cell-defined MGST3-high M2-like state was reflected at the disease level, we structured bulk validation into three complementary layers: disease-context comparison in four discovery plaque cohorts, cross-cohort generalization of the MGST3-high M2 signature across seven bulk cohorts, and independent validation of MGST3-associated mitochondrial programs. Across the four discovery datasets, ssGSEA showed that M2-like scores were consistently higher in AS than in control samples (all P < 0.05; **Figure 7A**), indicating reproducible enrichment of M2-like macrophage programs in diseased plaques. State deconvolution further showed increased resident-like M2 macrophages (SMD = 1.19, 95% CI 0.34-2.04, P = 0.0063) and total M2 macrophage proportions (SMD = 0.82, 95% CI 0.20-1.43, P = 0.0093) in disease (**Figure 7B**), supporting AS-associated expansion of M2-related macrophage states rather than a score-only effect. Across seven bulk cohorts, the single-cell-derived MGST3-high M2 signature was positively associated with M2-like proportions (meta rho = 0.47, 95% CI 0.32-0.58, P < 1E-8, I2 = 10.7%; **Figure 7C**), suggesting that it tracks M2-like macrophage abundance at the tissue-transcriptome level with low cross-cohort heterogeneity. Leave-one-out analysis confirmed that this association remained stable after sequential exclusion of individual cohorts (rho = 0.41-0.50; **Figure 7D**), indicating that it was not driven by a single dataset. Finally, in the independent cohort GSE111782 (n = 18), MGST3 expression was positively correlated with the MGST3-high M2 signature and mitochondrial functional modules (**Figure 7E**), providing external support for the association between MGST3 and M2-like mitochondrial programs. Together, these analyses connect the single-cell-defined MGST3-high M2-like state to AS-associated M2 expansion and mitochondrial functional programs in bulk plaque transcriptomes. Detailed results are provided in **Supplementary Table S11**.

**Figure 7:**
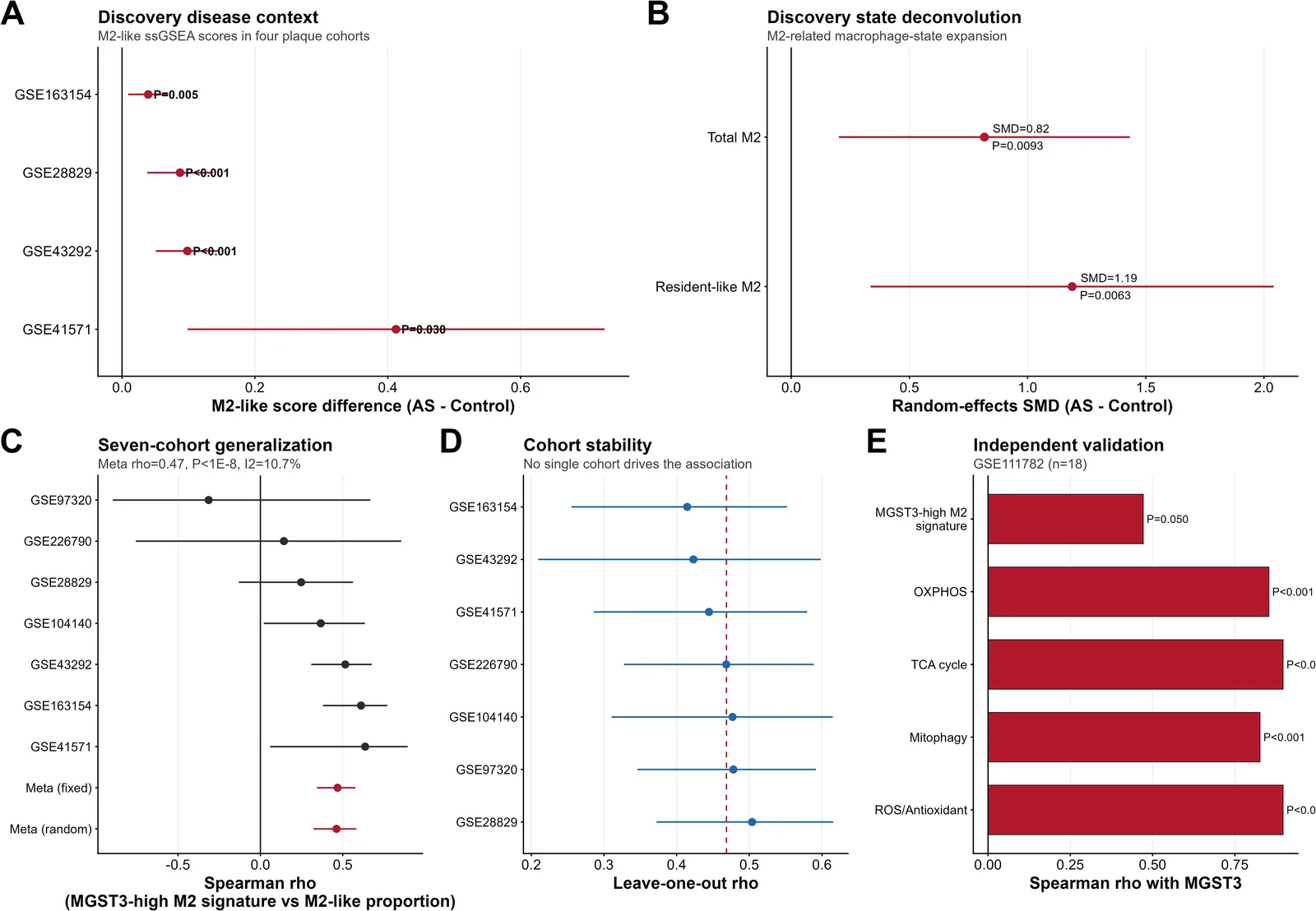
Disease-level bulk validation of the MGST3-high M2-like state. **(A)** ssGSEA M2-like scores in four discovery datasets. **(B)** Random-effects meta-analysis of resident-like and total M2-like proportions in disease. **(C)** Seven-cohort meta-analysis of the MGST3-high M2-like signature versus M2-like proportion. **(D)** Leave-one-out sensitivity analysis. **(E)** Independent cohort GSE111782: correlation of MGST3 with the MGST3-high M2-like signature and mitochondrial modules.

### MGST3-associated mitochondrial health

Having linked the MGST3-high M2-like state to disease-level M2 expansion and bulk-transcriptomic validation, we next directly tested whether MGST3 was associated with mitochondrial health within M2-like macrophages. We defined a composite MitoHealth score by integrating OXPHOS, TCA cycle, mitochondrial translation, and antioxidant modules and subtracting the inflammatory score, thereby capturing the balance between mitochondrial metabolic integrity and inflammatory activation. MitoHealth was consistently higher in MGST3-high than in MGST3-low M2-like cells across all three AS samples (paired P = 0.0415; **Figure 8A**), indicating a more favorable mitochondrial functional state at the sample level. At the single-cell level, MitoHealth correlated positively with MGST3 expression (rho = 0.40; **Figure 8B**), supporting a continuous relationship between MGST3 abundance and mitochondrial integrity. This association was reproducible in an independent single-cell processing pipeline, with concordant correlations for OXPHOS (0.26 vs 0.23), antioxidant defense (0.43 vs 0.69), mitophagy (0.12 vs 0.17), and inflammatory modules (-0.26 vs -0.32) between the main and rerun pipelines (**Figure 8C**). At the gene level, MGST3 was positively co-expressed with mitophagy receptor genes (**Figure 8D**) and antioxidant genes (**Figure 8E**), linking MGST3 to mitochondrial quality control and oxidative-stress buffering. The independent bulk cohort GSE111782 further confirmed positive co-expression between MGST3 and mitochondrial genes, including OXPHOS- and TCA-related genes (**Figure 8F**). Together, these sample-level, cell-level, pipeline-level, gene-level, and external-cohort analyses support MGST3 as a robust marker of mitochondrial health, quality control, and antioxidant defense in M2-like macrophages. Detailed results are provided in **Supplementary Table S12**.

**Figure 8:**
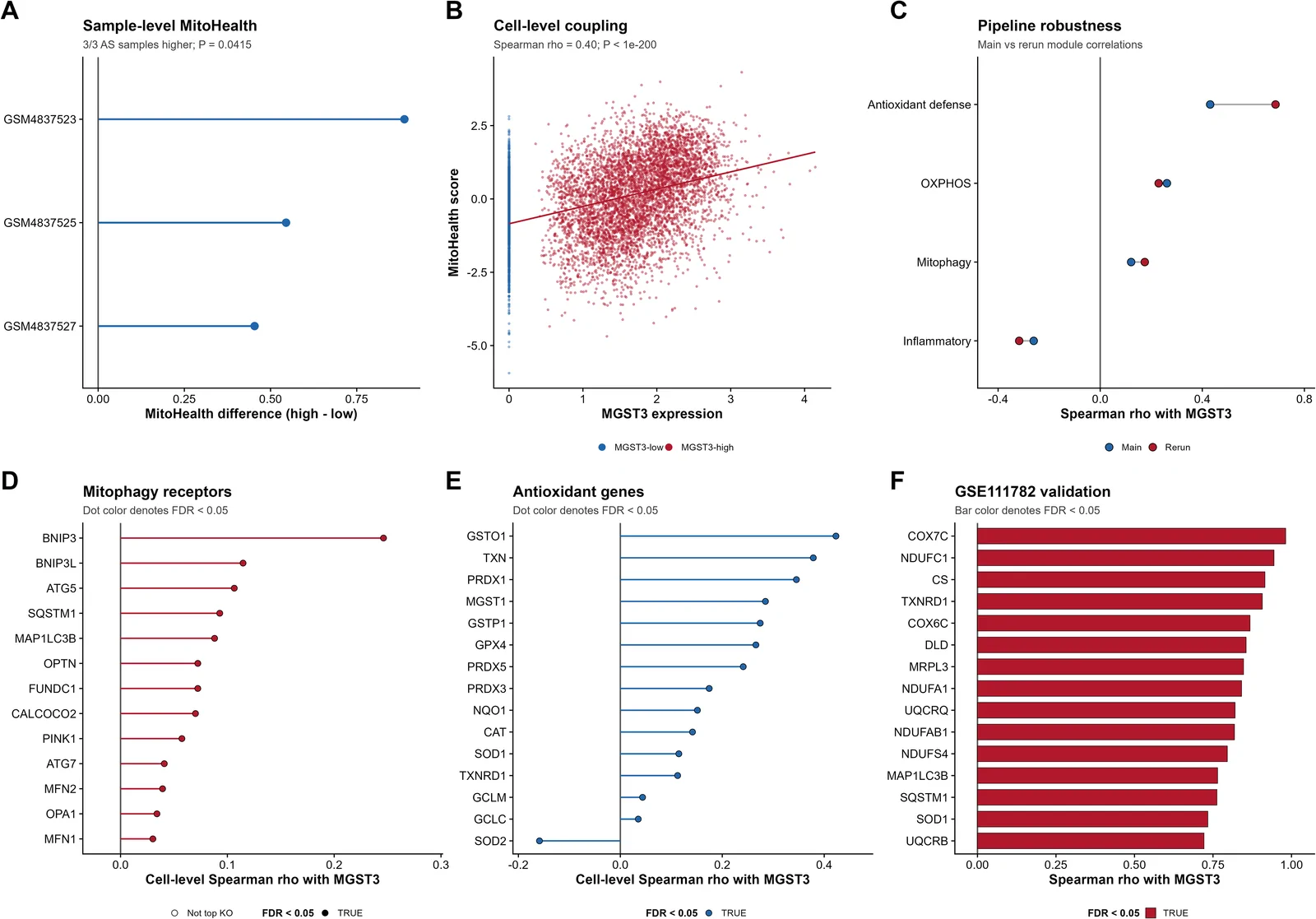
MGST3-associated mitochondrial health. **(A)** Paired MitoHealth difference between MGST3-high and MGST3-low M2-like cells. **(B)** Cell-level correlation between MGST3 and MitoHealth. **(C)** Pipeline robustness between the main and rerun single-cell analyses. **(D)** Cell-level co-expression of mitophagy receptors with MGST3. **(E)** Cell-level co-expression of GSH/antioxidant genes with MGST3. **(F)** Independent cohort GSE111782: mitochondrial gene co-expression with MGST3.

### Single-cell mediation and integrated protective model

Having established that MGST3 is associated with enhanced mitochondrial health in M2-like macrophages, we next asked whether this mitochondrial state connects MGST3 to the protective macrophage phenotype observed in AS plaques. Single-cell mediation analysis was performed using MitoHealth as the mediator between MGST3 and pro-atherogenic macrophage phenotypes. Sample-cluster bootstrap analysis showed significantly negative indirect effects of MGST3 through MitoHealth for inflammatory score (IE = -0.301, 95% CI -0.313 to -0.290), M1 score (IE = -0.173, 95% CI -0.176 to -0.166), and atherogenic composite score (IE = -0.197, 95% CI -0.228 to -0.178; all bootstrap P < 0.001; **Figures 9A–C**). These results indicate that higher MGST3 is associated with reduced inflammatory activation, lower M1-like polarization, and attenuation of broader pro-atherogenic macrophage programs partly through improved mitochondrial health. The integrated model (**Figure 9D**) summarizes this protective cascade, in which the MGST3-high M2-like state maintains mitochondrial metabolic integrity and antioxidant capacity, restrains inflammatory and M1-like activation, and supports a macrophage program consistent with plaque protection. Detailed results are provided in **Supplementary Table S13**. Together, these findings provide a mechanistic synthesis linking MGST3, mitochondrial preservation, and M2-like macrophage-mediated protection in atherosclerosis.

**Figure 9:**
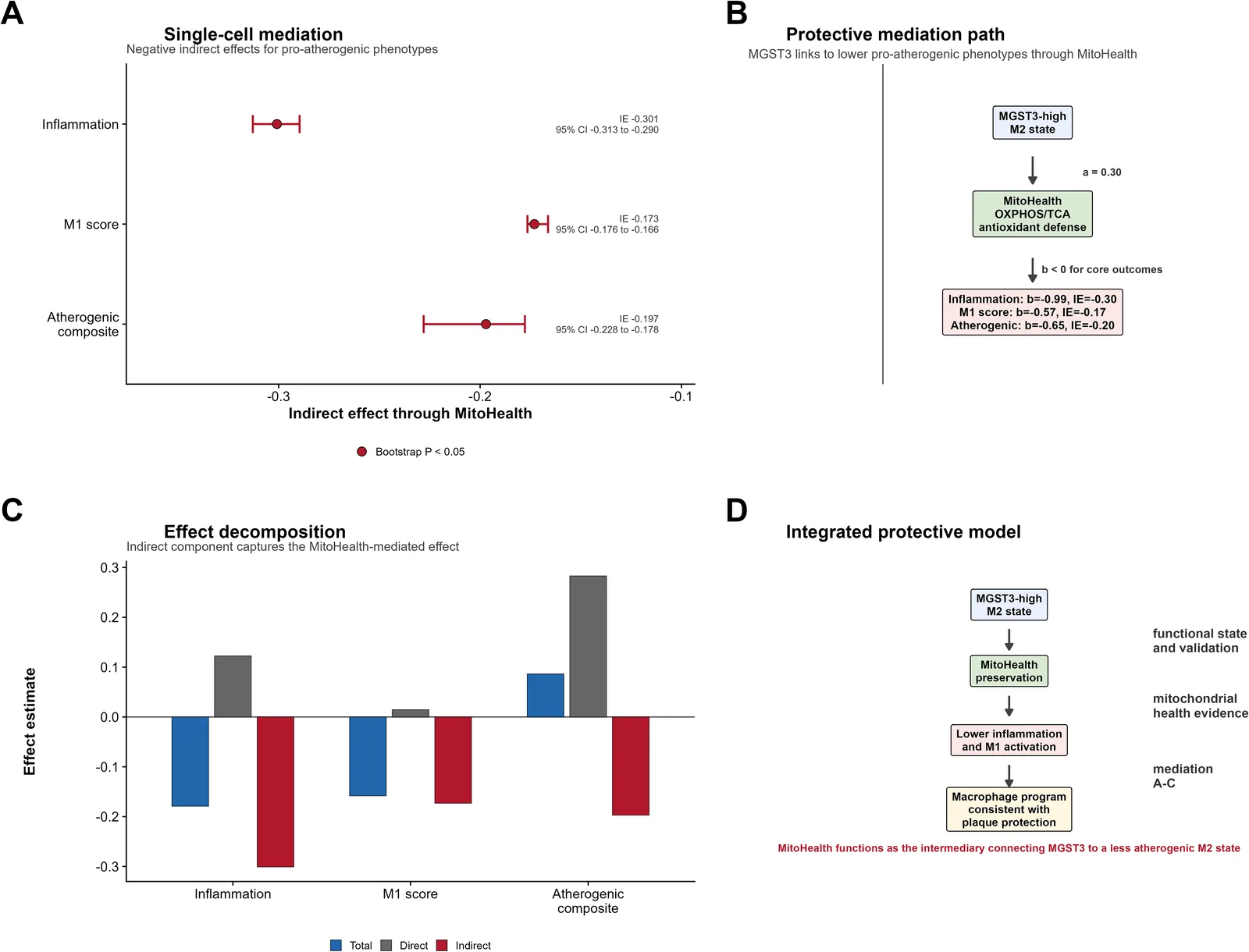
Single-cell mediation and integrated macrophage model. **(A)** Indirect effects of MGST3 on inflammatory, M1-like, and atherogenic composite scores through MitoHealth (cluster bootstrap by sample). **(B)** Mediation path with path coefficients. **(C)** Total, direct, and indirect effect decomposition. **(D)** Integrated model summarizing the MGST3-high M2-like state -> MitoHealth -> lower inflammation and M1-like activation -> a plaque-protective macrophage program.

### PheWAS analysis

PheWAS was performed for MGST3 across two phenotype sets (**Figures 10A, B**) using 17,361 binary and 1,419 quantitative phenotypes from the AstraZeneca PheWAS Portal. Because PheWAS identifies associations between genetically predicted gene expression and diseases or traits, we applied a stringent significance threshold of P < 1E-8. MGST3 showed no significant associations with the surveyed phenotypes under this criterion. These findings indicate an absence of detectable associations in the analyzed PheWAS resource, but they should not be interpreted as evidence that pharmacological modulation of MGST3 would be safe.

**Figure 10:**
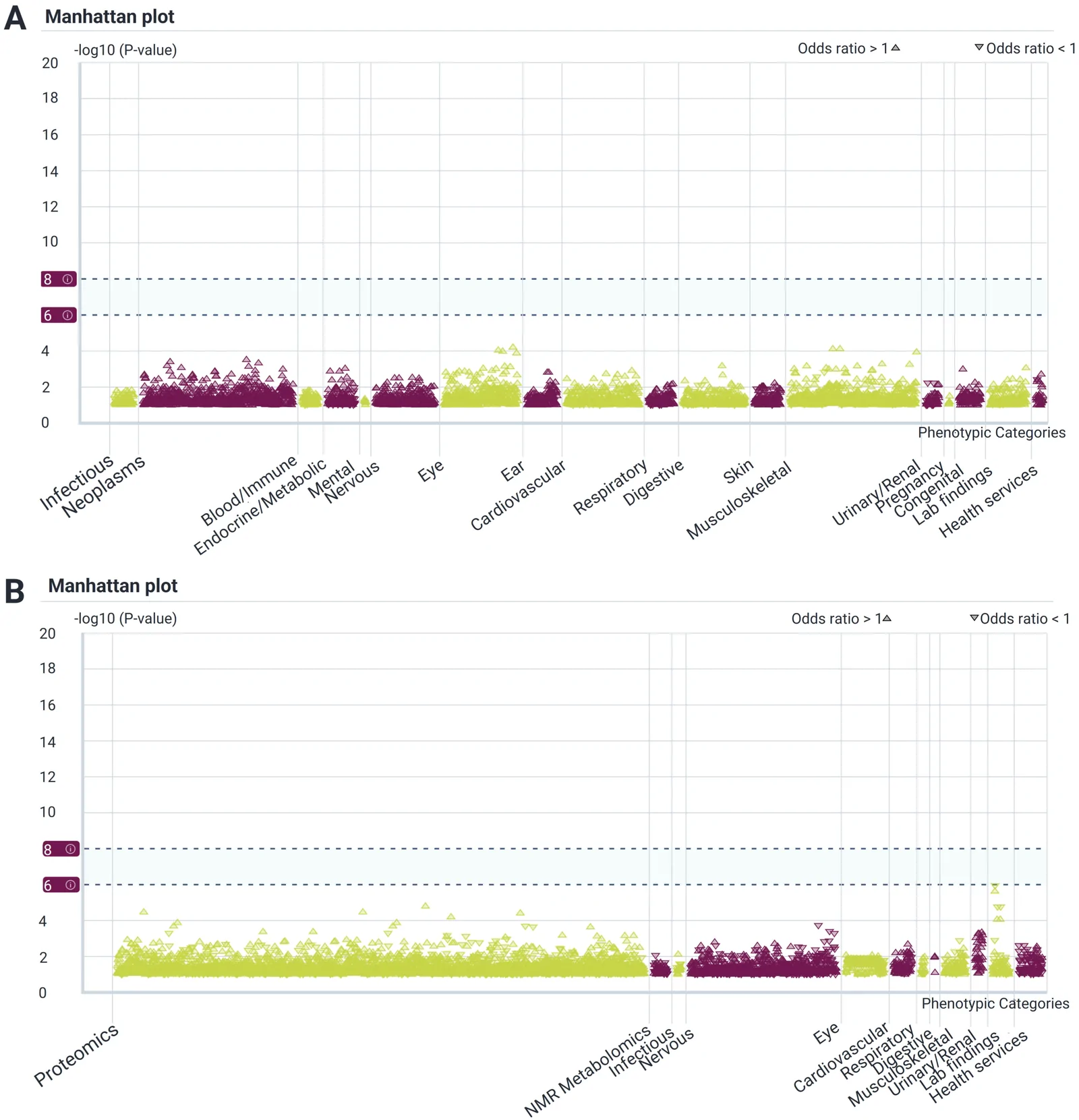
PheWAS of MGST3. Manhattan plots illustrating broad phenotype associations for MGST3 in the AstraZeneca PheWAS Portal across 17,361 binary phenotypes **(A)** and 1,419 quantitative phenotypes **(B)**. The x-axis represents individual phenotypes, and the y-axis indicates −log10(P). No significant associations were observed for any phenotype in either set at the predefined P < 1E−8 threshold. These results indicate no detectable associations in the analyzed PheWAS resource and should not be interpreted as evidence of pharmacological safety.

### Plaque-level MGST3 validation and *in vitro* functional support in M2-like macrophages

IHC staining showed reduced MGST3 immunoreactivity in AS plaques compared with paired macroscopically intact non-plaque arterial tissues, supporting lower MGST3 protein abundance in the plaque regions examined (**Figure 11A**). Consistently, RT-qPCR demonstrated significantly lower MGST3 mRNA expression in AS samples than in non-plaque arterial samples (P < 0.05; **Figure 11B**). Western blotting further confirmed reduced MGST3 protein expression in AS tissues relative to paired non-plaque arterial tissues, with GAPDH-normalized densitometry showing a statistically significant decrease (P < 0.05; **Figure 11C**). To test the functional role of MGST3 in macrophages, we performed RT-qPCR after MGST3 knockdown in M2-polarized THP-1 macrophages. Compared with siNC, siMGST3 significantly reduced MGST3 expression, confirming efficient knockdown, and decreased the M2-associated markers MRC1 (0.70-fold), CD163 (0.72-fold), and IL10 (0.72-fold), while increasing the pro-inflammatory markers IL1B (1.56-fold) and TNF (1.52-fold; all P < 0.001; **Figure 11D**). In the same validation panel, MGST3 knockdown reduced mitochondrial and antioxidant markers, including NDUFC1 (0.64-fold), COX7A2 (0.72-fold), GPX4 (0.68-fold), and SOD2 (0.63-fold; all P < 0.001; **Figure 11D**). Together, plaque-level and *in vitro* validation support reduced MGST3 expression in AS lesions and a role for MGST3 in maintaining the anti-inflammatory and mitochondrial-health-associated phenotype of M2-like macrophages. Detailed results are provided in **Supplementary Table S14**.

**Figure 11:**
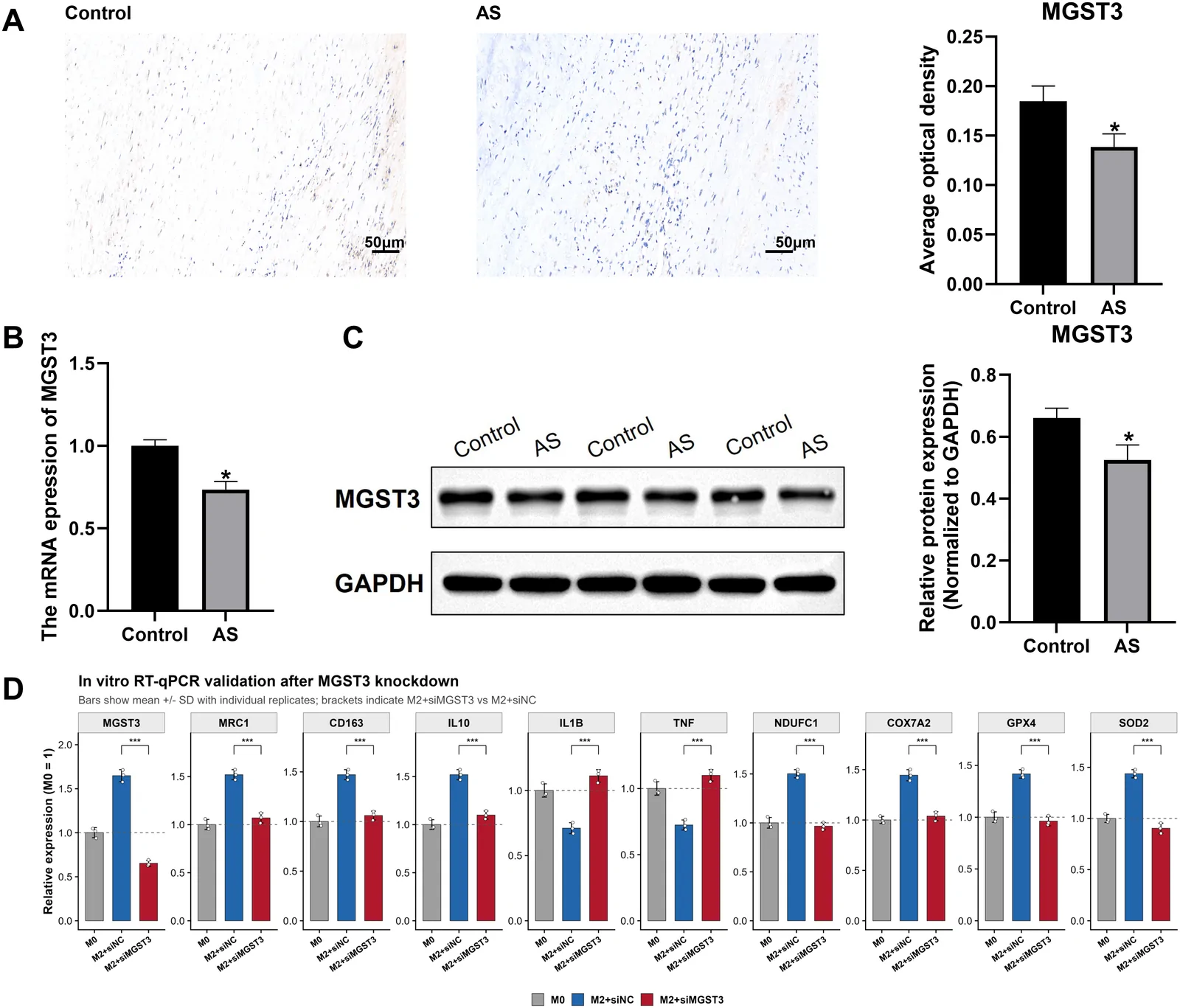
Plaque-level MGST3 validation and *in vitro* functional support in M2-like macrophages. **(A)** IHC staining of MGST3 in paired macroscopically intact non-plaque arterial tissue and AS plaque regions re-cropped from original whole-slide images (scale bar: 200 μm). **(B)** RT-qPCR analysis of MGST3 mRNA expression in non-plaque arterial tissues versus AS samples. Data are presented as mean ± SEM. **(C)** Western blot analysis of MGST3 protein expression normalized to GAPDH. The bar graph shows densitometric quantification. Data are presented as mean ± SEM. P < 0.05 for all. **(D)** Integrated *in vitro* RT-qPCR validation of M2-associated/pro-inflammatory markers (MGST3, MRC1, CD163, IL10, IL1B, TNF) and mitochondrial/antioxidant markers (NDUFC1, COX7A2, GPX4, SOD2) in THP-1-derived M2-like macrophages after siNC or siMGST3 transfection. Data are shown as mean ± SD relative to M0 (M0 = 1; n = 3); brackets indicate M2+siMGST3 versus M2+siNC comparisons; *P < 0.05, 8***P < 0.001 (unpaired t-test).

## Discussion

This study establishes an integrative framework linking MGST3 to macrophage mitochondrial protection in AS. Previous transcriptomic studies have identified plaque-associated genes, mitochondrial signatures, or immune-cell infiltration patterns mainly from bulk expression profiles (42–44). Although these studies provided useful candidate markers, they generally could not determine whether a prioritized gene was genetically supported, which plaque cell state expressed it, or how it might shape macrophage function. Unlike prior studies that mainly described macrophage polarization or mitochondrial dysfunction as separate features of AS plaques, our analysis links a specific mitochondrial gene, a defined M2-like macrophage state, and a functional mitochondrial-health program within the same disease context. By integrating bulk transcriptomic screening, genetic prioritization, single-cell macrophage-state mapping, mitochondrial mechanism analysis, cell-cell communication inference, disease-level validation, and *in vitro* perturbation, our study positions MGST3 as more than a differentially expressed transcript. Instead, MGST3 emerged as a glutathione-related mitochondrial gene associated with a distinct M2-like macrophage state characterized by preserved mitochondrial activity, reduced inflammatory activation, and potential plaque-protective communication. To our knowledge, this study provides one of the first integrative analyses to identify an MGST3-enriched M2-like macrophage state in AS plaques and to connect this state with mitochondrial health and anti-atherogenic macrophage programming.

Mechanistically, our findings suggest that MGST3 may help M2-like macrophages maintain functional stability under the oxidative and lipid-rich stress of atherosclerotic plaques. Macrophage mitochondrial dysfunction, ROS accumulation, and oxidized lipid stress are recognized contributors to inflammatory activation and plaque progression, whereas reparative macrophage states depend on preserved OXPHOS, TCA-cycle activity, redox balance, and mitochondrial quality control (15, 45, 46). In this context, the enrichment of OXPHOS, TCA-cycle, mitochondrial translation, ROS/antioxidant, and mitophagy-related programs in MGST3-high M2-like cells suggests a coordinated mitochondrial-health phenotype rather than isolated metabolic activation. This is biologically plausible because MGST3 belongs to the glutathione S-transferase family and may participate in glutathione-dependent detoxification of lipid peroxidation products (22, 47–49). Therefore, higher MGST3 expression may reduce oxidative lipid burden, preserve mitochondrial integrity, and help maintain an anti-inflammatory M2-like program within the plaque. By buffering lipid-peroxidation-derived oxidative injury, MGST3 may help preserve electron-transport-chain activity and mitochondrial quality-control capacity, thereby preventing mitochondrial stress from amplifying inflammatory macrophage activation. Importantly, this state should not be interpreted as a conventional anti-inflammatory M2 phenotype alone; in human plaques, macrophage programs are shaped by lipid loading, oxidative stress, mitochondrial metabolism, and local remodeling cues (15, 50). The MGST3-high M2-like program may therefore represent a stress-adapted macrophage state that preserves mitochondrial function and limits inflammatory escalation while remaining responsive to plaque remodeling signals. Consistently, MGST3-high M2-like cells showed lower inflammatory and M1-like activity, reduced pro-inflammatory transcription-factor activity, and predicted sensitivity of lipid and prostanoid metabolic networks to MGST3 perturbation. The single-cell mediation analysis should be interpreted as supporting this directionality at the statistical level: higher MGST3 was associated with higher MitoHealth, and higher MitoHealth was associated with lower inflammatory score, lower M1-like score, and lower atherogenic composite score. Rather than proving a direct causal chain, this analysis provides a statistical bridge between MGST3 expression, mitochondrial health, and reduced pro-atherogenic macrophage programs, thereby generating a testable mechanistic model for future functional studies.

At the plaque level, the MGST3-enriched M2-like state refines rather than simply repeats previous observations on macrophage heterogeneity in AS. Previous studies have shown that macrophage phenotypes in atherosclerotic plaques cannot be fully captured by a simple M1/M2 dichotomy (23, 51, 52). Both M1- and M2-associated markers may accumulate during lesion development, and single-cell studies have identified resident-like, foamy, inflammatory, and anti-inflammatory macrophage subsets within plaques. Therefore, the presence or expansion of M2-like macrophages in AS is not, by itself, unexpected. The distinctive contribution of the present study is the identification of an MGST3-enriched M2-like macrophage state that is increased in AS and is linked to mitochondrial health, antioxidant activity, reduced inflammatory activation, and plaque microenvironmental communication. This finding also helps explain the apparent discrepancy between decreased MGST3 expression at the whole-tissue level and increased MGST3 expression within M2-like macrophages. Bulk plaque transcriptomes reflect the average signal of multiple vascular, stromal, and immune cell populations, whereas single-cell analysis resolves a specific macrophage subset in which MGST3 is relatively enriched. Rather than contradicting the tissue-level decrease, the expansion of MGST3-enriched M2-like cells may indicate a compensatory protective cell state that emerges in response to oxidative, lipid-rich, and inflammatory stress within atherosclerotic plaques.

The biological relevance of this state is further supported by its communication profile, external validation, and experimental perturbation. Cell-cell communication analysis suggested that MGST3-high M2-like cells may act as an active signaling population within plaques through SPP1, MIF, CD99, MHC-II, and GALECTIN pathways. Because SPP1-associated plaque macrophage programs and MIF-CD74 signaling have been linked to context-dependent inflammatory, remodeling, and regulatory responses, these pathways should not be interpreted as uniformly protective (51, 53). Rather, they suggest that MGST3-high M2-like cells may integrate mitochondrial adaptation with context-dependent immune-regulatory and tissue-remodeling communication in the plaque microenvironment (32). Disease-level analyses supported this interpretation: M2-like scores were elevated across discovery cohorts, deconvolution indicated expansion of resident-like and total M2-like populations, and the MGST3-high M2 signature tracked M2-like proportions across seven bulk cohorts. The independent GSE111782 cohort supports the mitochondrial-program component of this model by showing positive co-expression between MGST3 and mitochondrial-health-related genes, but it should not be overinterpreted as direct proof of cellular-composition effects. Finally, *in vitro* MGST3 knockdown reduced M2 markers and mitochondrial/antioxidant transcripts while increasing pro-inflammatory transcripts, providing experimental support for the predicted MGST3-MitoHealth-M2 axis.

Several limitations should be considered without diminishing the central convergence of the evidence. The MR analysis used blood-derived eQTLs rather than plaque macrophage-specific eQTLs, and the public bulk cohorts differ in platform, plaque phenotype, and tissue composition; therefore, the genetic and cohort-level results should be interpreted as supportive rather than definitive plaque-cell-specific causality. In addition, scTenifoldKnk and mediation analyses are computational inference methods, and the top virtual-knockdown candidate genes were not independently validated by plaque-level qPCR. Because the central aim of the experimental validation was to test whether MGST3 itself supports the mitochondrial-health-associated M2-like state, the current *in vitro* experiments focused on MGST3 knockdown and representative M2-associated, inflammatory, mitochondrial, and antioxidant transcripts, but remained mainly transcript based. Direct animal-model evidence for MGST3 in atherosclerosis is also not yet available. Future studies should directly measure MGST3 enzymatic activity, lipid peroxidation, ROS production, OXPHOS function, mitophagy flux, macrophage-specific MGST3 localization in plaque tissue, and the *in vivo* effect of MGST3 modulation on plaque progression. Nonetheless, the agreement among genetic prioritization, single-cell state analysis, mitochondrial co-expression, disease-level validation, and MGST3 knockdown supports a coherent model in which MGST3 preserves mitochondrial health and restrains pro-atherogenic activation in M2-like macrophages during AS.

## Conclusions

Through integrative multi-omics, single-cell mediation, and functional validation, this study supports a model in which MGST3 is linked to reduced atherosclerotic risk by preserving mitochondrial health in M2-like macrophages. MGST3 is preferentially expressed in M2-like reparative macrophages, and high MGST3 expression is associated with an OXPHOS-oriented, antioxidant, and less inflammatory state in which MitoHealth statistically connects MGST3 to lower pro-atherogenic macrophage programs. These findings nominate the MGST3-MitoHealth axis as a candidate macrophage pathway for diagnostic and therapeutic investigation, pending direct activity-based, spatial, and *in vivo* validation.

## Data Availability

The original contributions presented in the study are included in the article/**Supplementary Material**. Further inquiries can be directed to the corresponding authors.
