# Supplementary material for: MGST3 defines a mitochondrial-health-associated M2 macrophage state in atherosclerosis: integrative multi-omics, single-cell mediation, and functional validation
Source: Front Immunol. 2026 Sep 17;17:1864957. doi: 10.3389/fimmu.2026.1864957 (PMC13627009; doi:10.3389/fimmu.2026.1864957)
Supplement: Supplementary file 1 [file Table1.docx]

**Supplementary Figure S1**. Immune cell infiltration differences between control and AS samples and their association with selected mitochondrial genes.

(A) Comparative analysis of immune cell infiltration between control and AS samples. AS samples showed increased infiltration of M0 macrophages and decreased infiltration of CD8 T cells and resting dendritic cells compared with controls. Detailed immune infiltration results are provided in **Supplementary Table S6**.

**(B)** Correlation analysis between M0 macrophage infiltration abundance and eight selected mitochondrial genes. PPIF, PMAIP1, GATM, and BCL2A1 were positively correlated with M0 macrophage infiltration, whereas MRPL33, MGST3, IMMP2L, and CPS1 showed negative correlations.
